# Effects of Assimilable Organic Carbon and Free Chlorine on Bacterial Growth in Drinking Water

**DOI:** 10.1371/journal.pone.0128825

**Published:** 2015-06-02

**Authors:** Xiaolu Liu, Jingqi Wang, Tingting Liu, Weiwen Kong, Xiaoqing He, Yi Jin, Bolin Zhang

**Affiliations:** 1 College of Biological Sciences and Technology, P. O. Box 162, Beijing Forestry University, Beijing 100083, China; 2 State Key Laboratory of Environmental Aquatic Chemistry, Research Center for Eco-Environmental Sciences, Chinese Academy of Sciences, P. O. Box 2871, Beijing, 100085, China; Instituto de Biologia, BRAZIL

## Abstract

Assimilable organic carbon (AOC) is one of the most important factors affecting the re-growth of microorganisms in drinking water. High AOC concentrations result in biological instability, but disinfection kills microbes to ensure the safety of drinking water. Free chlorine is an important oxidizing agent used during the disinfection process. Therefore, we explored the combined effects of AOC and free chlorine on bacterial growth in drinking water using flow cytometry (FCM). The initial AOC concentration was 168 μg.L^-1^ in all water samples. Without free chlorine, the concentrations of intact bacteria increased but the level of AOC decreased. The addition of sodium hypochlorite caused an increase and fluctuation in AOC due to the oxidation of organic carbon. The concentrations of intact bacteria decreased from 1.1×10^5^ cells.mL^-1^ to 2.6×10^4^ cells.mL^-1^ at an initial free chlorine dose of 0.6 mg.L^-1^ to 4.8×10^4^ cells.mL^-1^ at an initial free chlorine dose of 0.3 mg.L^-1^ due to free chlorine originating from sodium hypochlorite. Additionally, free chlorine might be more obviously affected AOC concentrations than microbial growth did. These results suggested that AOC and free chlorine might have combined effects on microbial growth. In this study, our results showed concentrations determined by FCM were higher than those by HPC, which indicated that some *E*. *coli* detected by FCM might not be detected using HPC in drinking water. The level of free chlorine might restrain the consumption of AOC by inhibiting the growth of *E*. *coli*; on the other hand, chlorination might increase the level of AOC, thereby increase the potential for microbial growth in the drinking water network.

## Introduction

With global industrialization and economization, aquatic pollution has become a problem worldwide [1]. As drinking water becomes more polluted, conventional water purification systems are rendered less effective in terms of removing pollutants [2], which decreases water quality and causes potential risks to human health. For example, waterborne disease outbreaks caused by pathogenic microorganisms are now commonplace [3, 4]. To ensure the safety of drinking water, microbial quality standards have been established globally. United States standards dictate that the heterotrophic plate count must be below 500 CFU.mL^-1^. The European Union directive states that for every 100 mL of drinking water, no total coliforms should be detected [5], and in China, heterotrophic plate counts in tap water must be lower than 100 CFU.mL^-1^, while no coliforms or fecal coliforms should be detected in 100 mL of water [6].

Conventional detection methods in aquatic microbiology have traditionally included a heterotrophic plate count (HPC) and spectrophotometry, but each method has limitations. Plating is slow and enables detection of less than 1% of the microorganisms present in natural environments [7], while spectrophotometry is more rapid (requiring only seconds per sample), but high detection limits and indirect counts restrict its application [8]. Some oligotrophs, thermophiles, and autotrophs are non-cultivable, and many bacteria enter a “viable but non-culturable (VBNC)” state when the growth environment (such as temperature and nutrition composition) changes. The VBNC state leads to inaccurate results when using HPC as a detection method [9]. Molecular detection methods based on the polymerase chain reaction (PCR) have been developed recently; these offer new approaches to the detection and recognition of pathogen DNA with the potential to obtain results in a much shorter time (hours) than possible with conventional methods. However, given the sensitivity of PCR, the greatest limitation of these methods is the inability to distinguish between live and dead cells, and a false positive may occur as a result of environmental contamination [10]. FCM used in microbiology since the late 1970s and now used commonly in aquatic microbiology, is highly accurate, rapid, and can identify components of interest. FCM used in combination with fluorescent stains reveals important information such as total cell concentration, bacterial viability, bacterial characteristics or bacterial identity in water samples. It can monitor changes of aquatic bacterial communities in drinking water systems by fast differentiating of dissimilar bacterial communities, and allowing accurate detection of even small changes in aquatic environments [11]. A real time flow cytometry approach allowed the collection of detailed, time-resolved information on complex processes [12]. The combination of FCM and pyrosequencing methods is a promising approach for future drinking water quality monitoring and for advanced studies on drinking water distribution pipeline ecology [13]. Now FCM is the standardised protocol proposed in the Swiss guideline for water analysis [14]. The microbial re-growth potential of drinking water is an important indicator of pathogenic microbial pollution. Although microbes are killed by disinfectants before water is transported to distribution systems and residual chlorine exists in the water network and the extremities of drinking water distribution systems, there are still several problems with household drinking water, such as increases in the number of heterotrophic bacteria [15]. Microbial growth also induces changes in turbidity, organic matters pollution, and nitrite, as well as metal corrosion and scaling [16].

Assimilable organic carbon (AOC) is one of the most important causes of microorganism re-growth in drinking water distribution systems; it is defined as the fraction of labile dissolved organic carbon that is more easily assimilated by microorganisms than other types of organic carbon. AOC consists of a broad range of low-molecular-weight organic carbon molecules, such as sugars, organic acids, and amino acids. It accounts for only a small fraction (0.1%-10%) of the total organic carbon (TOC) in water [17], but is considered one of the main indicators of biological water stability [18] because a high AOC concentration allows the re-growth of bacteria that have survived drinking water disinfection [19]. To control or prevent the re-growth of microorganisms, waterworks use chlorine, chloramine, ozone, and UV as disinfectants, with chlorination being the most common method in many countries. Van der Kooij (1992) found that drinking water is biologically stable when the AOC concentration is less than 10 μg.L^-1^ [20]. LeChevallier et al. (1996) found that in the presence of chlorine, AOC values range from 50 μg.L^-1^ to 100 μg.L^-1^ [21], and a recent study showed that at a chlorine level of 50 μg.L^-1^, bacteria may not re-grow, even at AOC concentrations of 100–150 μg.L^-1^ [22].

Some studies have reported relationships between AOC and microbial growth, AOC and chlorine, and between chlorine and microbial growth. However, these interactions (especially in drinking water) require further characterization. Thus, we explored the combined effects of AOC and free chlorine on bacterial growth in drinking water using a series of experiments. We added *Escherichia coli* to tap water at an initial concentration optimal for the FCM and produced simulated pathogen-contaminated drinking water samples. We then added various concentrations of sodium hypochlorite to simulate chlorination and investigated the combined impact of AOC and free chlorine on bacterial growth for 72 h. Our results provide guidelines for the removal of AOC and the addition of free chlorine to promote the safety of drinking water.

## Materials and Methods

### Bacterial strains and pre-cultivation

*Escherichia coli*, acquired from the China General Microbiological Culture Collection Center, was stored at -80°C before use. The cryo-culture was incubated in Luria-Bertani (LB) broth for 12 h at 37°C, after which the bacterial suspension was streaked onto a nutrient agar plate (Huankai, China) and incubated for 24 h at 37°C. Cells from a single colony were transferred with a loop into LB broth diluted 10-fold and incubated overnight at 37°C. Subsequently, cells were transferred into LB medium diluted 1000-fold (starting concentration: 5×10^3^ cells.mL^-1^) and incubated for 4 days at 30°C to be used as inoculum.

### Sampling and treatment

Drinking water samples were acquired from our laboratory in the Haidian District (Beijing), and water was flushed for 15 min before sampling. We added 1 L of water to three conical flasks with stoppers, and then added a solution of sodium thiosulfate (0.5 mol.L^-1^) to neutralize residual chlorine. Two samples were treated with sodium hypochlorite (1 mg.mL^-1^ free chlorine) at initial free chlorine concentrations of 0.3 mg.L^-1^ and 0.6 mg.L^-1^. The third sample was used as a control. We centrifuged pre-cultivated *E*. *coli* inoculum at 13,000 rpm for 5 min, discarded the supernatant, and added ultrapure water to remix the pellet. This process was then repeated twice. Finally, the *E*. *coli* suspension was added to water samples (initial concentration: 1×10^5^ cells.mL^-1^) and samples were incubated at 25°C in the dark. Lastly, we measured the concentrations of intact bacteria, free chlorine, and AOC at 0, 4, 12, 24, 36, 48, and 72 h.

### Preparation of carbon-free materials

Carbon-free glassware (bottles and vials) was prepared as described by Hammes and Egli [17]. All glassware was first washed with a common detergent and rinsed three times with deionized water. It was then rinsed again with deionized water and air-dried. Finally, bottles and vials were heated in a muffle furnace at 550°C for at least 3 h to burn off residual carbon compounds. Teflon-coated screw caps for the glassware were soaked in a 10% sodium persulphate solution at 60°C for 1 h, rinsed three times with deionized water, and air dried.

### AOC measurements

We determined AOC concentrations as described previously [23, 24]. The pasteurized and filtered water samples (15 mL) were inoculated with a bacterial AOC test inoculum (initial concentration: 1×10^4^ cells.mL^-1^), the suspensions were incubated at 30°C for 4 days in the dark (until stationary phase), and the resulting growth was measured with FCM (see below). We then used Eq 1 to determine the AOC concentration. The AOC test inoculum comprised the indigenous drinking water community and a commercial mineral water (Evian), and was incubated at 30°C for 2–3 days (Eq 1).

$$\text{AOC (}\mu \text{gC}{\text{. L}}^{\text{-1}}\text{)}=\frac{\text{net grown cells}{\text{. L}}^{\text{ -1}}}{\text{1}\times {\text{10}}^{\text{7}}\text{(cells. }\mu {\text{gC}}^{\text{-1}}\text{)}}$$ (1)

### Fluorescence staining and flow cytometry

Staining was performed as described previously [25]. For a working solution, we diluted SYBR Green I (SG) (Invitrogen, USA) 100-fold in anhydrous dimethylsulfoxide (DMSO), and added propidium iodide (PI, 30 mM) to a final PI concentration of 0.6 mM (SGPI). This working solution was stored at -20°C until use. We stained 1 mL of water sample with 10 μL SYBR Green I working solution for enumeration of total bacteria or 10 μL SGPI for enumeration of intact/damaged bacterial cells. Before analysis, samples were incubated in the dark at 37°C for 15 min and bacteria-counting beads (Invitrogen, USA) were added to each sample for calculation. Prior to FCM analysis, water samples were diluted with 0.22 μm filtered bottled water (Evian) to concentrations ranging from 1×10^5^ cells.mL^-1^ to 1×10^6^ cells.mL^-1^. We performed FCM using a FACSCalibur (BD Biosciences, USA) instrument with CellQuest software equipped with an argon laser emitting light at a fixed wavelength of 488 nm. In the flow cytometer, optical filters were set up so that PI was measured above 630 nm and SYBR Green I at 520 ± 10 nm. The trigger was set for the green fluorescence (520 nm) channel (FL1), and data were acquired on two-parameter dot plots of green fluorescence (520 nm) versus red fluorescence (630 nm); ultrapure water was used as sheath fluid. No compensation was used for any measurement.

### Data analysis

The average value, logarithm, standard deviation and coefficient of variance were calculated by Excel software (Microsoft, Redmond, Washington) spreadsheets and the figures were also drawn by the spreadsheets. The statistical analysis was performed using one-way ANOVAs with SPSS 12.0 (SPSS Taiwan Corp., Taiwan) and p < 0.05 was considered significant.

## Results

### A comparison of two detection methods for enumerating bacteria

**Fig 1:**
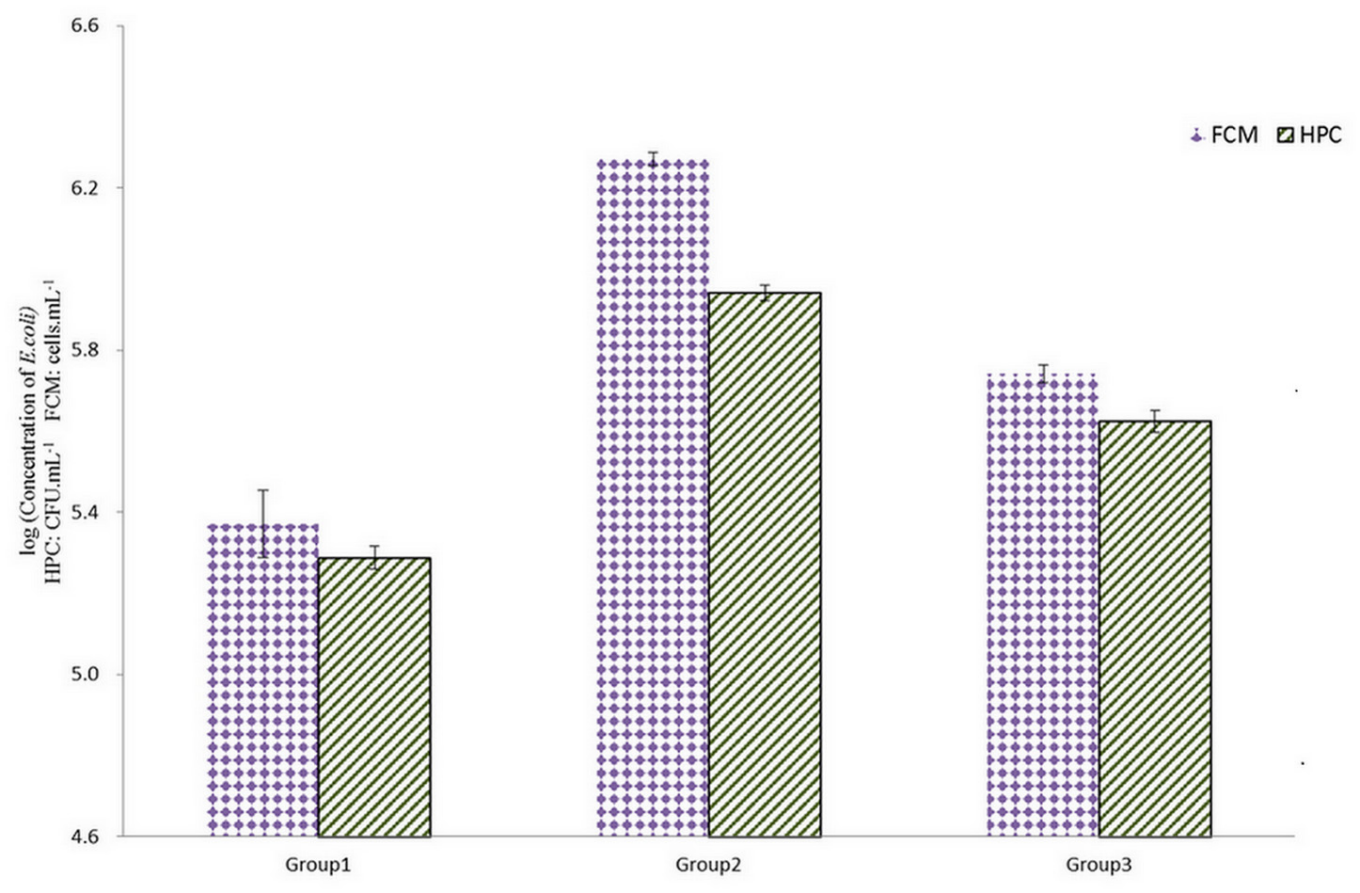
The results of two different methods to enumerate intact *Escherichia coli’*s concentration. SYPI stained cells were enumerated by flow cytometry for intact cell counts. The unit of FCM’s results is cells.mL^-1^ and the unit of HPC’s results is CFU.mL^-1^. All data points are average values for triplicate samples. Error bars indicate standard deviations. Group 1–3 were three groups of random *Escherichia coli* suspensions.

The general microbial quality of drinking water is normally monitored by HPC. This method has been used for more than 100 years and is recommended in drinking water guidelines. However, the HPC method is significantly handicapped because it is time-consuming and restricted to culturable bacteria [26]. We used the FCM and HPC to enumerate intact cells in the same *E*. *coli* suspension, and repeated this process three times. The results (Fig 1 and supplementary material) indicated that the intact cell counts determined by FCM were higher than those by HPC, meaning some VBNC *E*. *coli* detected by FCM might not be detected using HPC. However, the variable coefficients of FCM (7.4%, 7.8%, and 7.8%) were lower than those of HPC (10.2%, 11.9%, and 11.9%) (S1 File). Therefore, FCM combined with fluorescent dyes facilitated more consistent enumeration of bacteria than HPC.

### The relationship between microbial growth and free chlorine in water samples

**Fig 2:**
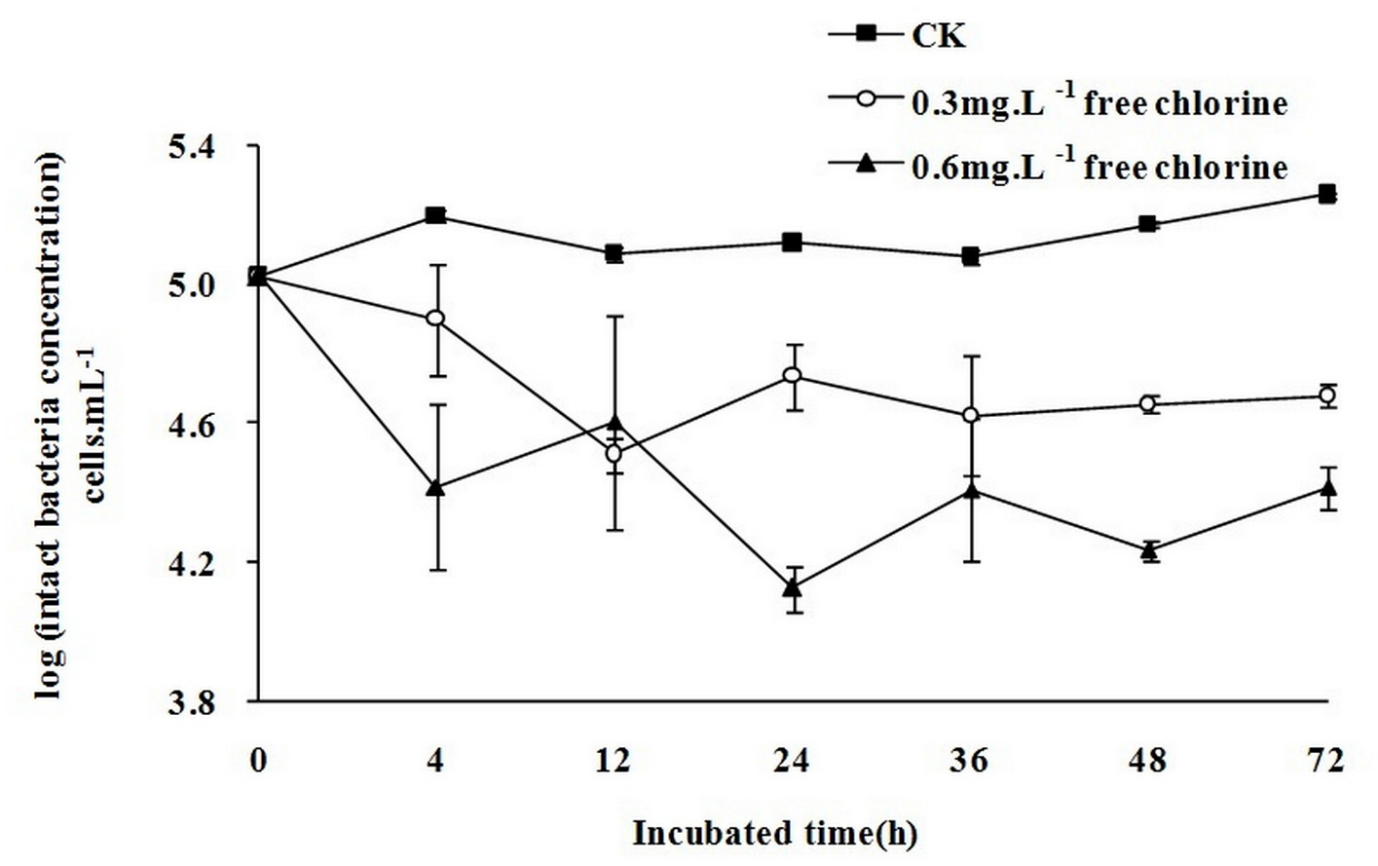
The variation tendency of intact bacteria concentration in 72h. All data points are average values for triplicate samples. Error bars indicate standard deviations. (CK means the initial free chlorine concentrations were 0 mg.L^-1^. The line with circles means the initial free chlorine concentrations were 0.3 mg.L^-1^ and the line with triangles means the initial free chlorine concentrations were 0.6 mg.L^-1^).

The correlation between disinfectant concentrations and percentage of intact cells was considered interesting as chlorine concentrations had been shown to be inversely proportional to cell survival [27]. Without free chlorine (Fig 2), the intact bacteria concentration in water samples increased from 1.1×10^5^ cells.mL^-1^ to 1.8×10^5^ cells.mL^-1^ over 72 h. When sodium hypochlorite was added to water samples, intact bacteria concentrations in the 0.6 mg.L^-1^ free chlorine sample decreased more rapidly than those in the 0.3 mg.L^-1^ free chlorine sample. Although intact bacterial concentrations fluctuated, they decreased relatively to the initial concentrations (peak concentrations of intact bacteria were 38.0%, 24.3%, and 51.7% of the initial concentrations). The final concentrations of intact bacteria (4.8×10^4^ cells.mL^-1^ in the 0.3 mg.L^-1^ free chlorine sample and 2.6×10^4^ cells.mL^-1^ in the 0.6 mg.L^-1^ free chlorine sample) were lower than the initial concentrations (1.1×10^5^ cells.mL^-1^). Lastly, the final concentration of intact bacteria in the 0.6 mg.L^-1^ free chlorine sample (2.6×10^4^ cells.mL^-1^) was lower than that in the 0.3 mg.L^-1^ free chlorine sample (4.8×10^4^ cells.mL^-1^).

### The relationship between AOC concentration and free chlorine in water samples

**Fig 3:**
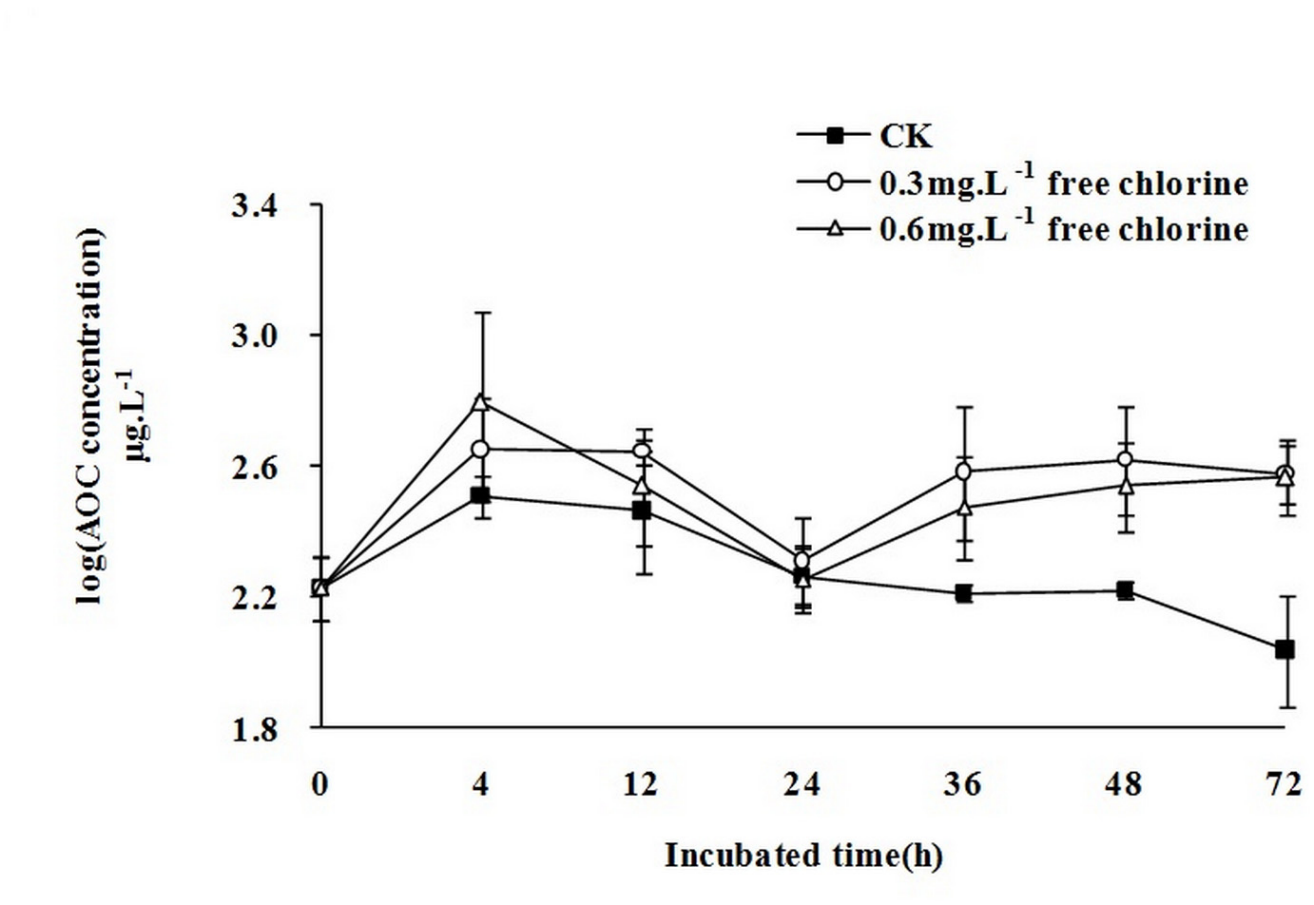
The variation tendency of AOC concentration in 72h. All data points are average values for triplicate samples. Error bars indicate standard deviations. (CK means the initial free chlorine concentrations were 0 mg.L^-1^. The line with circles means the initial free chlorine concentrations were 0.3 mg.L^-1^ and the line with triangles means the initial free chlorine concentrations were 0.6 mg.L^-1^).

AOC is a useful indicator to diagnose the potential of water quality deterioration by microbial regrowth or the biological stability of water. In all water samples, the initial AOC concentrations were 168 μg.L^-1^ (Fig 3). Upon addition of sodium hypochlorite, the concentrations fluctuated. In the 0.3 mg.L^-1^ free chlorine water sample, the AOC concentration increased sharply to 446 μg.L^-1^ in the first 4 h, stabilized, and then decreased to 204 μg.L^-1^ by 24 h. Subsequently, the AOC concentration of 72 h (376 μg.L^-1^) was lower than that of 48 h (416 μg.L^-1^), but not statistically significant (P>0.1). The AOC concentration trend in the 0.6 mg.L^-1^ free chlorine water sample was similar, but with several notable differences. The first peak value of the 0.6 mg.L^-1^ free chlorine water sample was 622 μg.L^-1^, which was higher than that of the 0.3 mg.L^-1^ free chlorine sample. After 12 h, the AOC concentration of the 0.6 mg.L^-1^ free chlorine sample was lower than that of the 0.3 mg.L^-1^ free chlorine sample, but not statistically significant (P>0.1). Without free chlorine, the AOC concentration increased to 322 μg.L^-1^ in the first 4 h and then decreased slowly to 109 μg.L^-1^ by 72 h. In short, our results showed the level of free chlorine might restrain the consumption of AOC by inhibiting the growth of *E*. *coli* (Figs 2 and 3).

### The combined effects of AOC and free chlorine on microbial growth in water

Without free chlorine (S1A Fig), a reduction in AOC (from 291 μg.L^-1^ to 109 μg.L^-1^) occurred together with an increase in cell numbers (from 1.2×10^5^ cells.mL^-1^ to 1.8×10^5^ cells.mL^-1^) at 12–72 h. In the 0.3 mg.L^-1^ free chlorine water sample (S1B Fig), the AOC concentration increased to 446 μg.L^-1^ during the initial 12 h, while the intact bacteria concentration decreased to 3.3×10^4^ cells.mL^-1^. Subsequently, the AOC concentration decreased to 204 μg.L^-1^ at 24 h and then increased till 72 h, while intact cell concentrations showed the opposite trend. In the 0.6 mg.L^-1^ free chlorine water sample (S1C Fig), the AOC concentration increased to 622 μg.L^-1^ by 4 h and then decreased at 4–12 h, while the intact bacteria concentration decreased to 2.6×10^4^ cells.mL^-1^ by 4 h and then increased at 4–12 h. After 12 h, the trends in AOC concentration and microbial growth were similar. S1B and S1C Fig showed in drinking water free chlorine might increase the concentration of AOC, too. Our results demonstrated that AOC and free chlorine had the combined effects on bacterial growth. On the one hand, free chlorine could inhibit the consumption of AOC; on the other hand, chlorination would significantly increase the level of AOC, thereby increasing the potential for microbial growth in the pipe network.

## Discussion

Researchers have estimated that <1% of the microorganisms present in natural environments can be detected by conventional plating [7]. Therefore, the conventional HPC method of enumerating bacteria may lead to underestimation of their concentrations [28]. Many studies have demonstrated that, when combined with fluorescent dyes, VBNC bacteria could be detected by FCM [29, 30]. Siebel (2008) found that HPC total counts were on average two orders of magnitude lower than those determined by FCM [26]. In a recent research the conventional HPC results were compared with the FCM intact cell concentration values [31]. And a weak correlation (R^2^ = 0.18, n = 38) was observed between HPC (at 22°C) and intact cell concentrations, similar to reports in previous studies [25]. It could be explained by the often described phenomenon, that less than 1% of drinking water bacteria are cultivable on conventional agar plates [25]. In addition, Mezule and coworkers [32] demonstrated evidence of the presence of VNBC bacterial state, in both drinking water and biofilms, thus indicating further limitations in the HPC method.

FCM can also be used to determine AOC concentrations. Conventional AOC analysis was originally developed by Van der Kooij et al. (1982) [20] and later updated by others [33, 34]. Due to their different metabolic capabilities, both the *Pseudomonas fluorescens* strain P-17 and *Spirillum* strain NOX are commonly used as test organisms to determine AOC concentrations. These strains are widespread in aquatic environments and can consume most biodegradable organic matters, but the conventional method is tedious, time-consuming, and labor-intensive. Moreover, we were unsure whether pure cultures would reliably reflect the AOC concentration of complex natural waters. With this in mind, we used the AOC determination method established by Hammes (2005) [17]. This method is used to determine the net growth of the total indigenous microbial community of water samples cultivated at 30°C for 3–4 days (until stationary phase).

In drinking water distribution systems, both AOC and disinfectants have important effects on bacterial growth. Disinfectants such as ozone, chlorine, and chloramine can destroy the structures of organic matters macromolecules and transform them into AOC, which induces bacterial re-growth [35]. A previous study designed to understand the impact of disinfection on the microbiology of reclaimed water in the distribution system revealed that chlorination will significantly increase the level of biodegradable organic matters including AOC, thereby increasing the potential for microbial regrowth in the pipe network [36]. In our experiment, we used distribution system water and various concentrations of sodium hypochlorite to detect changes in AOC concentrations over 72 h. These changes showed four distinct periods. Between 0–4 h, AOC concentrations increased sharply. Between 4–24 h AOC concentrations decreased and between 24–48 h AOC concentrations increased slowly. Finally, between 48–72 h, AOC concentrations were either stable or decreased slowly. In our opinion, these phenomena would be caused by these reasons: between 0–4 h, some organic matters were oxidized into AOC by free chlorine; between 4–24 h, AOC was oxidized into small-molecule compounds, such as CO_2_ and H_2_O, by free chlorine; between 24–48 h, some organic matters which were not oxidized were resolved into AOC slowly; between 48–72 h, the remaining AOC was oxidized into small-molecule compounds. Without free chlorine AOC concentrations also increased between 0–4 h. This phenomenon may be caused by combined chlorine, which was not neutralized by sodium thiosulfate in the water sample. Between 4–72 h, AOC concentrations decreased slowly because total chlorine was neutralized by sodium thiosulfate and the AOC was consumed only by bacteria. Although we used drinking water and performed our experiment over a longer period of time, our results are similar to those of Fang [37]. In this previous report, upstream raw water from the Huangpu River (treated with a sand filter column) was used and the experiment was conducted for 24 h. We used a 72 h period because drinking water may take 2–3 days to be transported from waterworks (after disinfection) to households in Beijing.

The use of FCM to detect changes in intact bacteria concentrations indicated that, in the presence of free chlorine, intact bacteria concentrations were reduced to 45.5% (0.3 mg.L^-1^ free chlorine water sample) and 24.6% (0.6 mg.L^-1^ free chlorine water sample) of their initial concentrations. In the absence of free chlorine, the final concentration of intact bacteria was 170.8% of the initial concentration; these increases in intact bacteria concentrations were accompanied by a reduction in the AOC concentration (S1 Fig). However, both bacteria and AOC concentrations increased between 0–4 h (S1A Fig) and 24–36 h (S1C Fig). Increases in AOC may be caused by free chlorine if it has a greater effect on AOC concentrations than bacterial growth did. Meanwhile, intact microbes could increase under a relatively low AOC concentration without free chlorine. However, upon addition of free chlorine, although the AOC concentration increased four-fold (S1B Fig), the number of intact microbes still tended to decrease. Therefore, free chlorine is the main factor that affects the number of intact bacteria.

Our data indicate that 0.3 mg.L^-1^ free chlorine can reduce the number of microbes in drinking water by ~2×10^4^ cells.mL^-1^. According to Cheng [38], the viable bacteria concentration in drinking water is ~10–100 CFU.mL^-1^ before transporting into distribution systems. Theoretically, a free chlorine dose of 0.3 mg.L^-1^ can eliminate a certain fraction of bacteria. However, Cheng also showed that the rate of samples containing bacteria exceeded 28% in 155 Beijing tap water samples between 2006 and 2007. The total number of bacteria in finished water differed among the seven waterworks; however, bacterial removal was generally incomplete [38]. Surviving bacteria re-grow in tap water in the presence of residual chlorine and increasing AOC concentrations. Therefore, these factors may lead to microbial contamination of drinking water.

## Conclusion

In this study, concentrations determined by FCM were higher than those by HPC, which indicated that some VBNC *E*. *coli* detected by FCM might not be detected using HPC in drinking water. Our results showed the level of free chlorine might restrain the consumption of AOC by inhibiting the growth of *E*. *coli*, on the other hand, chlorination might increase the level of AOC, thereby increasing the potential for microbial regrowth in the drinking water network.

## Supporting Information

S1 File Average concentration of intact *E*.*coli* by FCM and HPC. (XLSX)

S1 Fig The relation between microbial growth and AOC concentration. (A) Without free chlorine; (B) The initial free chlorine concentration was 0.3 mg.L^-1^; (C) The initial free chlorine concentration was 0.6 mg.L^-1^. (All data points are average values for triplicate samples. The line with squares means the change of microbial growth and the line with diamonds means the change of AOC concentrations).
(TIF)
