# Supplementary figures and images for: Effects of Assimilable Organic Carbon and Free Chlorine on Bacterial Growth in Drinking Water
Source: PLoS One. 2015 Jun 2;10(6):e0128825. doi: 10.1371/journal.pone.0128825 (PMC4452771; doi:10.1371/journal.pone.0128825)

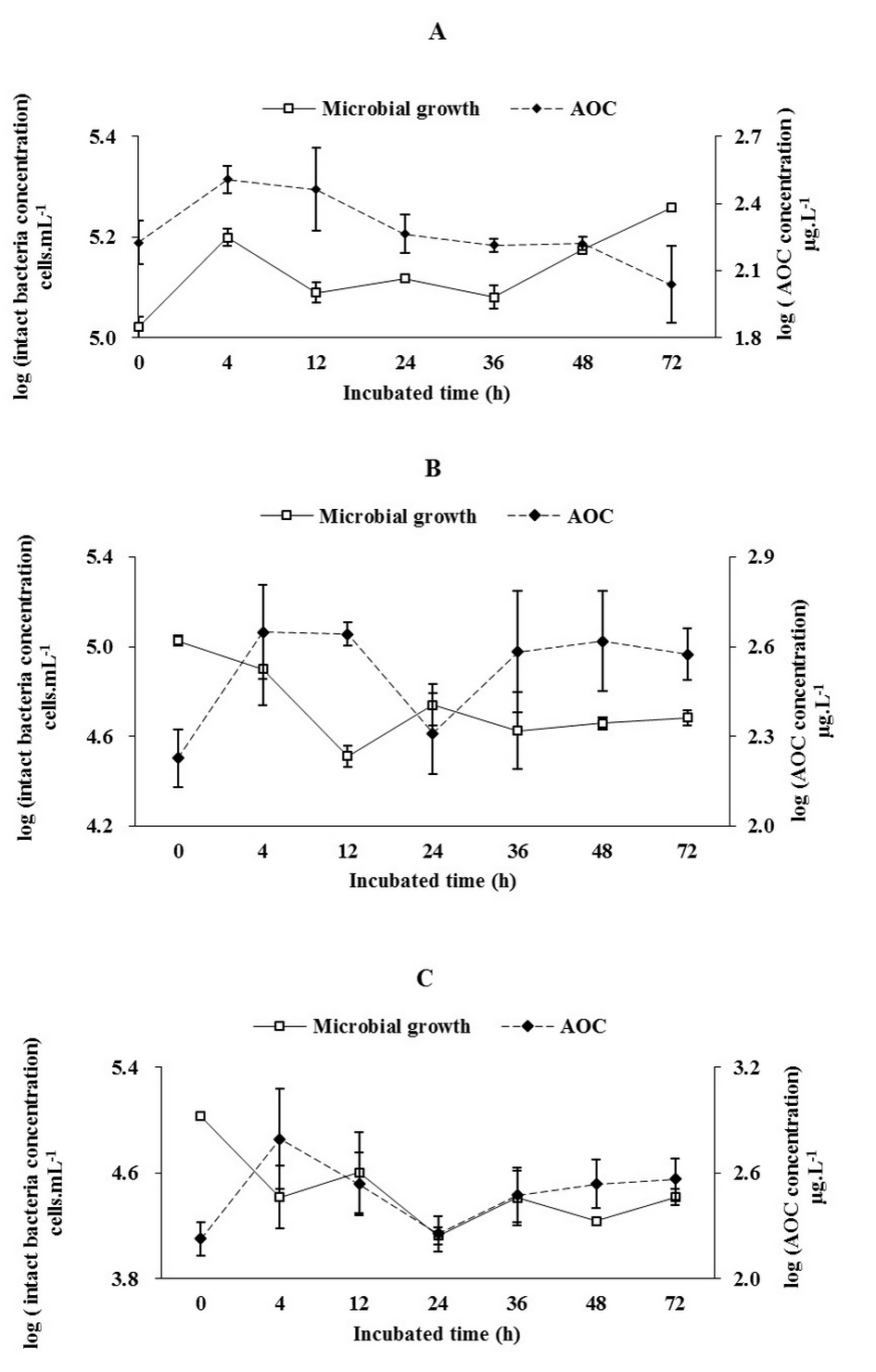

Supplement: S1 Fig — (A) Without free chlorine; (B) The initial free chlorine concentration was 0.3 mg.L-1; (C) The initial free chlorine concentration was 0.6 mg.L-1. (All data points are average values for triplicate samples. The line with squares means the change of microbial growth and the line with diamonds means the change of AOC concentrations). (TIF) [file pone.0128825.s002.tif]
